# Research progress on microRNA in gout

**DOI:** 10.3389/fphar.2022.981799

**Published:** 2022-10-20

**Authors:** Jing Xie, Cuixia He, Yue Su, Yuzhou Ding, Xingyu Zhu, Yuanyuan Xu, Jiaxiang Ding, Huan Zhou, Hongju Wang

**Affiliations:** ^1^ Clinical Trials Center, The First Affiliated Hospital of Bengbu Medical College, Bengbu, Anhui, China; ^2^ School of Pharmacy, Bengbu Medical College, Bengbu, Anhui, China; ^3^ School of Public Foundation, Bengbu Medical College, Bengbu, Anhui, China

**Keywords:** microRNA, gout, cellular signaling pathway, treatment, biomarker

## Abstract

Gout is a common form of arthritis caused by the deposition of sodium urate crystals in the joints and tissues around them. MicroRNAs (miRNAs) are noncoding RNAs that have been shown to be involved in regulating the pathogenesis of gout through multiple cellular signaling pathways, which may be potential targets for the treatment of gout. In this review, we systematically discuss the regulatory roles of related miRNAs in gout, which will provide help for the treatment of gout and miRNAs is expected to become a potential biomarker for gout diagnosis.

## Introduction

Gout is a kind of metabolic disease caused by the disorder of purine metabolism, which causes an increase in uric acid in blood, leading to the formation and deposition of monosodium urate (MSU) crystals in joints and other tissues. With the improvement of living standards and changes in lifestyle, the prevalence of gout has increased significantly in recent years, with an incidence of 0.58–2.89 per 1,000 person-years, causing great health damage to patients (Dehlin et al., 2020). The prevalence of gout is also increasing in developed countries compared to developing countries (Clebak et al., 2020; Singh and Cleveland, 2021). Patients with gout often have metabolic abnormalities and so are also prone to hypertension, hyperglycemia, hyperlipidemia and other conditions. Gout can also increase the incidences of chronic kidney disease and cardiovascular disease, seriously affecting human health (Edwards, 2008). Gout is related to age and sex. The affected patients are mainly middle-aged and elderly, and the incidence of gout is higher in males than in females (Dehlin et al., 2020). The inflammatory mechanism of gout is not fully understood, but it is widely believed that the NOD-like receptor family, pyrin domain-containing 3 (NLRP3) inflammasome and Toll-like receptor (TLR) signaling pathway play important roles. MSU crystals deposited in joints are phagocytosed and recognized by monocytes/macrophages, activate the NLRP3 inflammasome and promote the generation of mature interleukin (IL)-1β through the NLRP3 and TLR/nuclear factor-κB (NF-κB) signaling pathways. Activated IL-1β is secreted out of cells and then causes the recruitment and infiltration of inflammatory cells such as neutrophils and the release of more inflammatory mediators, promoting the occurrence and progression of the inflammatory response in gout (Narang and Dalbeth, 2020; Dalbeth et al., 2021). Studies have shown that a variety of microRNAs (miRNAs) are involved in regulating the pathogenesis of gout. For example, miR-223-3p and miR-22-3p reduce the production of IL-1β by targeting NLRP3, thereby alleviating the inflammatory response of gout (Wang et al., 2021).

MiRNAs are a class of single-stranded noncoding RNAs that mainly negatively regulate gene expression. MiRNAs were first discovered in *Caenorhabditis elegans* (Lee et al., 1993; Wightman et al., 1993). They are ubiquitous in eukaryotes, and thousands of miRNAs expressed in human cells have been identified (Papanagnou et al., 2016). MiRNAs play a role at the posttranscriptional level and participate in cell proliferation, apoptosis and other life processes (Hwang and Mendell, 2007). Abnormal expression of miRNAs can cause inflammatory diseases, including gout. For example, when miR-155 is overexpressed in cells, it promotes the production of excessive inflammatory cytokines and triggers an inflammatory response in patients with gout (Jin et al., 2014). In recent years, new miRNAs and signaling pathways have been discovered in gout, which may provide new ideas for the diagnosis and treatment of clinical diseases. This paper systematically reviews the relevant literature on the regulatory role of miRNAs in the pathophysiology of gout to provide a reference for the subsequent exploration and research of miRNAs.

## Overview of gout

In the 2018 edition of the European League Against Rheumatism (EULAR) updated evidence-based expert recommendations on the diagnosis of gout, the clinical manifestations of gout patients are divided into preclinical (asymptomatic hyperuricemia and asymptomatic MSU crystal deposition) and clinical gout [gout attack, critical zone gout and chronic gouty arthritis (CGA)] (Richette et al., 2020). The traditional natural course of gout includes asymptomatic hyperuricemia, acute onset, interictal and CGA.

The enzyme product of purine metabolism is uric acid. If the concentration of serum uric acid in the human body exceeds the saturation point of 6.8 mg/dl, uric acid deposition will occur, which will induce the onset of gouty arthritis (GA) (Mandell, 2008; Liu et al., 2015). The hyperuricemic phase is the early stage of gout, and approximately 10% of patients will eventually develop gout (Miao et al., 2009). At this stage, the focus of treatment is to control the uric acid in the blood to prevent blood uric acid levels from reaching or exceeding saturation and precipitating crystals, causing AGA (Keller and Mandell, 2021).

AGA is the second phase in gout patients. The body usually has no symptoms, but the first metatarsophalangeal joint, knee, ankle, or other joints may suddenly develop severe pain. During this period, patients may develop fever and joint swelling accompanied by severe pain, preventing them from sleeping normally and seriously affecting their sleep and quality of life (Mandell, 2008; Seow et al., 2020; Dalbeth et al., 2021). Predisposing factors include a high-purine diet, alcohol intake (Danve et al., 2021), the environment and other diseases (Dubreuil et al., 2013; Wu et al., 2022).

After the first gout attack, there is a high probability of a second, a third and so on. AGA then progresses to the gout attack intermittent period. MSU crystals continue to deposit around the joints and in the synovial tissue even during the intervals after attacks. Most patients relapse within 1–3 years of the first attack, with increasing frequency, prolonged duration and shorter intermission periods (Mandell, 2008; Dalbeth et al., 2021). If not treated, some patients with gout will continue to experience attacks, and the accumulation of excess uric acid in the body will not only be limited to within the joints but will begin to deposit slowly in the subcutaneous tissue and the CGA. At this stage, chronic joint swelling and pain will develop, which may lead to joint destruction, tophi and uric acid kidney stones (Edwards, 2008; Keller and Mandell, 2021).

## Overview of microRNAs

MiRNAs are endogenous noncoding RNAs consisting of approximately 20 nucleotides that inhibit messenger RNA (mRNA) translation by specifically binding to the 3′ untranslated region (UTR) of the target mRNA. As negative regulators, miRNAs play a specific role in inhibiting the translation of target genes at the posttranscriptional level (Dong et al., 2013). Extracellular miRNAs can be transported through extracellular vesicles, or secreted in the form of protein-miRNA complexes after assembly with specific proteins such as lipoproteins (Mori et al., 2019). It has been found that miRNAs are not only involved in the occurrence and development of cardiovascular diseases and malignant tumors (Lin and Gregory, 2015; McManus and Freedman, 2015) but also widely involved in the regulation of inflammatory diseases, including GA. For example, in the exploration of osteoarthritis (OA), miR-582-3p can reduce the secretion of pro-inflammatory cytokines and inhibit the apoptosis of chondrocytes (He et al., 2020). MiR-221-5p can directly inhibit IL-1β and regulate the pathogenesis of AGA (Li et al., 2021a). In general, as an important regulator of gene expression, miRNAs play a role in the pathogenesis of gout, which deserves our in-depth exploration.

## The functional role of microRNAs in gouty arthritis

Gout is a common inflammatory arthritis that is mainly caused by the accumulation of MSU crystals in tissues. MiR-142-3p, miR-155, miR-192-5p and many other miRNAs have been found to be abnormally expressed in GA and play important roles in the occurrence and development of GA (Table 1).

**TABLE 1:** MicroRNA associated in gout.

miRNAs	Location	Expression and upregulated/downregulated	Target gene(s)/pathway	Result	PMID
miR-142-3p	Human chromosome 17	MSU-stimulated THP-1 cells and MSU-treated male C57BL/6 mouse ankle joint tissue↑	ZEB2↓/NF-κB↑	IL-1β, IL-6, TNF-α↑	35239453
miR-146a	Human chromosome 5	Bone marrow-derived macrophages from MSU-stimulated B6 wild-type mice↑	TRAF6, IRAK1↓, TLR4/MyD88/NF-κB↓	NLRP3, TNF-α, IL-1, IL-6↓	31773674
miR-155	Human chromosome 21	Synovial fluid mononuclear cells in patients with AGA and MSU-induced C57BL gout mouse model↑	SHIP-1↓	TNF-α, IL-1β↑	24708712
miR-192-5p	Human chromosome 11	Serum of GA patients and synovial tissue and synovial fluid of MSU-treated male C57BL/6 mice↓	EREG↑	IL-1β, IL-6, TNF-α↑	34715618
miR-221-5p	Human chromosome X	AGA patient serum↓	IL-1β↑	IL-8, TNF-α↑	33201565
miR-223-3p/miR-22-3p	Human chromosome X	MSU-stimulated mouse air pouch synovium and myristin sodium acetate-treated THP-1 cells↓	NLRP3↑	IL-1β↑	33650318
miR-302b	Human chromosome 4	Serum of GA patients and MSU-treated THP-1 cells and mouse air pouch↑	IRAK4/NF-κB, EphA2/caspase-1↓	IL-1β↓	29482609
miR-488	Human chromosome 1	Peripheral blood leukocytes in patients with GA↓	IL-1β↑	IL-8, TNF-α↑	28915828
miR-920	Human chromosome 12	Peripheral blood leukocytes in patients with GA↓	IL-1β↑	IL-8, TNF-α↑	28915828

### MiR-142-3p

MiR-142-3p is located on human chromosome 17 and can play an important role as an anti-inflammatory or proinflammatory gene in inflammation-related diseases (Hu and Wang, 2016; Zhang et al., 2020a). For example, miR-142-3p is involved in the regulation of OA (Gao et al., 2019), rheumatoid arthritis (RA) (Renman et al., 2021) and GA (Bohatá et al., 2021). Wang et al. (2016) found that the expression of miR-142-3p was reduced in mice with OA and had a targeted regulatory role with high mobility group box 1 (HMGB1). Upregulation of miR-142-3p expression inhibited chondrocyte apoptosis and inflammation. MiR-142-3p was also found to be aberrantly expressed in RA patients compared to healthy controls (Renman et al., 2021). Most recently, Bohatá et al. (2021) detected the expression of miR-142-3p in the plasma of normal uric acid controls, hyperuricemia and gout patients, and the results showed that the expression of miR-142-3p was upregulated in hyperuricemia and gout patients.

### MiR-146a

MiR-146a is located on human chromosome five and mouse chromosome 11. MiR-146a was first proven to be involved in the regulation of innate immunity. Studies suggest that the imbalance of miR-146a expression is closely related to the pathogenesis of autoimmune diseases and inflammatory diseases and has anti-inflammatory effects in a variety of pathogeneses (Iborra et al., 2012; Garo and Murugaiyan, 2016). It is reported that miR-146a was highly expressed in peripheral blood mononuclear cells (PBMCs) and the synovium of patients with RA (Pauley et al., 2008; Stanczyk et al., 2008). Additionally, miR-146a deficiency leads to a significant increase in the expression of proinflammatory cytokines in macrophages in diabetic nephropathy and enhances the severity of the inflammatory response (Bhatt et al., 2016). Dalbeth detected the expression levels of miR-146a in PBMCs of acute and intermittent GA patients, hyperuricemia patients and normouricemia patients. The results showed that the expression of miR-146a was significantly increased in patients with gout in the intermittent period, suggesting that miR-146a may play a role in the intermittent period and participate in the negative regulation of gout inflammation (Dalbeth et al., 2015).

### MiR-155

The sequence of miR-155 is highly conserved in different species, and the miR-155 genes of humans, mice, and chickens are located on chromosomes 21, 16, and 1, respectively. MiR-155, as a multifunctional miRNA, is involved in a variety of disease processes, including cancer and inflammatory diseases. For an instance, miR-155 is highly expressed in a variety of malignancies, including colon cancer (Mashima, 2015; Li et al., 2018). Elmesmari et al. (2016) found that miR-155 was expressed at significantly higher levels in the peripheral blood and monocytes of RA patients than in controls and could promote the accumulation of inflammatory cells in the synovial membrane to cause disease by regulating the expression of chemokines and proinflammatory chemokine receptors. Experimental studies showed that mice with knockout of the miR-155 gene were completely protected from arthritis induced by collagen stimulation (Blüml et al., 2011). The study found that miR-155 was upregulated in synovial macrophages of RA patients, leading to downregulation of Src homology 2-containing inositol phosphatase 1 (SHIP-1) and increased production of proinflammatory cytokines (Kurowska-Stolarska et al., 2011). Jin et al. (2014) found that the expression of miR-155 was increased *in vitro* and *in vivo* in the GA model, and miR-155 also inhibited the expression level of SHIP-1, resulting in the upregulation of proinflammatory cytokines.

### MiR-192-5P

MiR-192-5p is a conserved miRNA located on human chromosome 11 that is expressed in the liver and is involved in the regulation of liver diseases such as chronic hepatitis B and acute liver injury (Roy et al., 2016; Nielsen et al., 2018). Zhou et al. (2018) found that miR-192-5p was upregulated in the plasma and tissues of pancreatic cancer patients but not in plasma exosomes. It is reported that miR-192-5p is downregulated in RA, and it can attenuate the inflammatory response by targeting Ras-related C3 botulinum toxin substrate 2 (RAC2) (Zheng et al., 2020). Zhang et al. (2020b) found that miR-192 plays an important role in polarization in macrophages. The role of miR-192-5p in GA has also attracted attention. By RT‒qPCR and ELISA analysis, An and Yin (2021) found that the expression of miR-192-5p in the serum of GA patients was significantly downregulated compared with healthy controls, while the expression of epiregulin (EREG) was significantly upregulated. MSU-induced joint damage and inflammation were attenuated when miR-192-5p was upregulated.

### MiR-221-5p

MiR-221-5p is located on chromosome X and is aberrantly expressed in RA synovial fibroblasts (Pandis et al., 2012). Additionally, miR-221 was found to be overexpressed in RA synovial tissue and patient serum. Downregulation of miR-221 induced a decrease in the expression of proinflammatory cytokines and induced apoptosis (Yang and Yang, 2015). In a recent study, Li et al. (2021a) detected the expression of miR-221-5p in the serum of patients with AGA and normal controls by RT‒PCR and found that the former expressed a significantly lower level than the latter. Through cellular experiments, they found that overexpression of miR-221-5p can promote the decreased expression of tumor necrosis factor-α (TNF-α), IL-8 and IL-1β, revealing that miR-221-5p inhibits the pathogenesis of AGA by targeting IL-1β.

### MiR-223-3p

MiR-223-3p is derived from a gene located on the X chromosome. MiR-223 was expressed in the human hematopoietic system (Chen et al., 2004), synovium and peripheral T lymphocytes from RA patients (Fulci et al., 2010; Shibuya et al., 2013). Bauernfeind et al. (2012) found that miR-223 is highly expressed in macrophages and can target and inhibit the expression of NLRP3, demonstrating the negative regulation between miR-223 and NLRP3. Moreover, Haneklaus’ team also found that miR-223 can inhibit the activity of NLRP3 and reduce the level of IL-1 by targeting the 3′ UTR of NLRP3 (Haneklaus et al., 2012). In recent years, miR-223-3p was found to be downregulated in murine myocarditis and may be involved in the regulation of hepatitis, myocarditis and other inflammatory diseases by regulating the expression of the NLRP3 inflammasome (Chen et al., 2020; Jimenez Calvente et al., 2020). Wang et al. (2021) found that miR-223-3p was significantly downregulated in MSU-stimulated mouse pouch synovium and phorbol myristate acetate-treated THP-1 cells compared to controls. When miR-223-3p was overexpressed in both, the expression of NLRP3 was significantly downregulated. It was further revealed that miR-223-3p can directly inhibit the expression of NLRP3, thereby reducing the inflammatory effect of gout.

### MiR-302b

MiR-302b is expressed from the miR-302/367 gene cluster located on human chromosome 4 (Gao et al., 2015). MiR-302b is specifically expressed in embryonic stem (ES) cells and is involved in the regulation of the ES cell cycle (Subramanyam et al., 2011). MiR-302b has also been shown to be widely involved in other biological processes. For example, miR-302b expression was significantly downregulated in osteosarcoma cell lines and clinical tumor tissues, and it can inhibit osteosarcoma cell migration and invasion by targeting runt-related transcription factor 2 (RUNX2) (Xie et al., 2017). Ma and Zhou (2020) found that miR-302b was downregulated in breast cancer patient tissues and cell lines by qRT‒PCR, which may predict poor prognosis in breast cancer patients. MiR-302b regulates inflammatory responses in respiratory bacterial infections (Zhou et al., 2014). In recent years, Ma et al. (2018) detected the expression of miR-302b in the serum of MSU-treated THP-1 cells, mouse air sacs and GA patients and found that it is highly expressed in all three and is involved in the regulation of related inflammatory responses.

### MiR-488 and miR-920

MiR-488 is located on chromosome 1 and has been shown to be aberrantly expressed in a variety of tumors, acting as an oncogene. For example, the expression of miR-488 is reduced in tissues and cell lines of tongue squamous cell carcinoma, and overexpression of miR-488 can inhibit tumor cell invasion (Shi et al., 2018). Compared to normal chondrocytes, miR-488 expression was reduced in chondrocytes of OA and involved in the development of hose cells (Papanagnou et al., 2016). MiR-920 is located on chromosome 12 and is downregulated in patients with osteoporosis and can bind to the 3′ UTR of homeobox gene A7 (HOXA7) mRNA to promote the osteogenic differentiation of human bone mesenchymal stem cells (Zha et al., 2020). MiR-920 is also involved in the regulation of glioblastoma cell development (Cong et al., 2020). There were few studies on miR-488 and miR-920 in GA until Zhou et al. (2017) found that the expression levels of both were significantly reduced in the peripheral blood leukocytes of GA patients. Overexpression of miR-488 and miR-920 promoted the downregulation of IL-1β, IL-8, and TNF-α in MSU-treated THP-1 cells. They further found that miR-488 and miR-920 could bind to the 3′ UTR of IL-1β to target and inhibit its production. These results suggest that miR-488 and miR-920 may play important regulatory roles in GA inflammation.

## The regulatory mechanism of microRNAs in gouty arthritis

**FIGURE 1:**
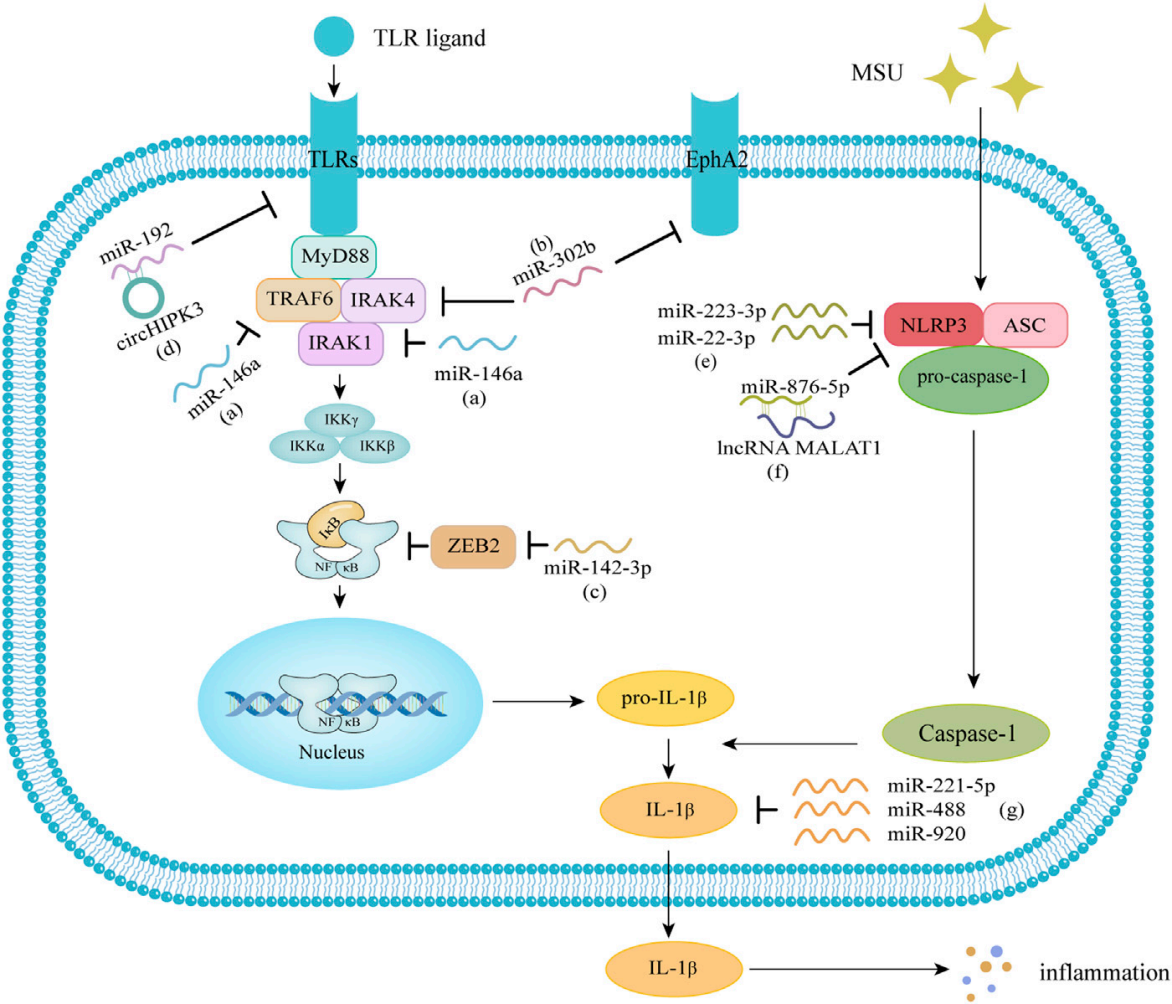
Molecular mechanisms of miRNA in gout. **(A)** miR-146a downregulates the levels of IL-1β, IL-6, TNF-α, and the NLRP3 inflammasome through the TLR/NF-κB signaling pathway. **(B)** miR-302b can negatively regulate the transcription and maturation of IL-1β by targeting IRAK4 and EphA2. **(C)** miR-142-3p can target and negatively regulate ZEB2, regulate NF-κB signaling, and lead to increased expression of IL-1β, IL-6, and TNF-α. **(D)** circular RNA circHIPK3 can act as a molecular sponge to bind miR-192, regulate the expression of TLR4. **(E)** miR-223-3p and miR-22-3p can directly inhibit the expression of NLRP3 to reduce the release of IL-1β. **(F)** lncRNA MALAT1 can reduce the inflammatory response through the miR-876-5p/NLRP3 pathway. **(G)** miR-221-5p, miR-488 and miR-920 can interact with the 3′ UTR of IL-1β and can target and inhibit IL-1β. MiRNA, microRNA; IL-1β, interleukin-1β; IL-6, interleukin-6; TNF-α, tumor necrosis factor-α; NLRP3, NOD-like receptor family, pyrin domain-containing 3; TLR, Toll-like receptor; NF-κB, nuclear factor-κB; IRAK4, IL-1 receptor-associated kinase 4; EphA2: Ephrin type-A receptor 2; ZEB2, zinc finger E-box binding homeobox 2; lncRNA, long noncoding RNA; MALAT1, metastasis-associated lung adenocarcinoma transcript 1.

The pathogenesis of GA is complex, and MSU deposited in joints can induce inflammatory cytokines such as IL-1β to mediate inflammatory responses through TLRs signaling pathway, NLRP3 signaling pathway and other cell signal transduction pathways. The TLRs signaling pathway releases pro-IL-1β under stimulation, and through the NLRP3 inflammasome signaling pathway, activated caspase-1 catalyzes the maturation of pro-IL-1β into IL-1β, which in turn triggers an inflammatory response. A variety of miRNAs are involved in regulating the above signaling pathways and may play an important role in the pathogenesis of GA (Figure 1).

### MicroRNAs and the toll-like receptor/molecule myeloid differentiation factor 88/nuclear factor-κB signaling pathway

TLRs are widely expressed in immune cells and have the ability to activate immune cells. TLRs can mediate the activation of the downstream NF-κB signaling pathway through the molecule myeloid differentiation factor 88 (MyD88) pathway. Research confirmed that MSU can act as an endogenous danger signal to activate the TLR cell signal transduction pathway and that the TLR signaling pathway is involved in the occurrence and development of GA. MSU crystals cooperate with free fatty acids or lipopolysaccharides as ligands to stimulate TLR2 and TLR4 on the surfaces of macrophages. The activated TLRs can recruit MyD88, successively activate IL-1 receptor-associated kinase (IRAK), etc., and then activate the NF-κB signaling pathway (Narang and Dalbeth, 2020; Dalbeth et al., 2021). It has been reported that circular RNA circHIPK3 can act as a molecular sponge to bind miR-192, regulate the expression of TLR4, and promote inflammation in GA (Lian et al., 2021). MiR-142-3p can target and negatively regulate zinc finger E-box binding homeobox 2 (ZEB2), regulate NF-κB signaling, and lead to increased expression of IL-1β, IL-6, and TNF-α, activating the inflammatory response in GA (Lu et al., 2022). Zhang et al. (2018) found that miR-146a KO mice had more severe GA and that the production and secretion of TNF receptor-associated factor 6 (TRAF6), IRAK1, and NLRP3 were increased compared with those in wild-type mice. Chen et al. (2019a) found that miR-146a can alleviate inflammation in rats with acute arthritis through the TLR4/MyD88/NF-κB signaling pathway. Ma et al. (2018) demonstrated that miR-302b can inhibit NF-κB signaling by targeting IRAK4 and negatively regulate the transcription and maturation of IL-1β, thereby regulating related inflammation. The TLR4/NF-κB pathway is closely related to GA and plays an important role in the occurrence and development of GA. Therefore, targeting this pathway through miRNA may provide a new direction for the diagnosis and treatment of GA.

### MicroRNAs and the NOD-like receptor family, pyrin domain-containing 3 inflammasome pathway

NLRP3 is one of the most studied and characterized multiprotein inflammatory complexes and consists of the NLRP3 protein, the apoptosis-associated speck-like protein (ASC), and pro-caspase-1 protease. NLRP3 is an important mediator of the host immune response and can be activated by MSU, which in turn regulates the maturation of IL-1 through further activation of caspase-1, causing inflammation. MiRNAs are considered to be important regulators of the NLRP3 inflammasome and are involved in its posttranscriptional regulation (de Zoete et al., 2014; Ozaki et al., 2015). Recently, Zamani et al. (2020) listed several miRNAs associated with NLRP3 regulation, including miR-146a, miR-155 and miR-223. Wang et al. (2021) confirmed that the direct target of miR-223-3p and miR-22-3p is NLRP3, and they can directly inhibit the expression of NLRP3 to reduce the release of IL-1β, thereby reducing the inflammatory effect of gout. Wang (2021) revealed that tripterine can regulate macrophage polarization through the miR-449a/NLRP3 axis to alleviate GA. Total glucoside (TGP) reduced MSU-induced activation of the NLRP3 inflammasome, and the overexpression of metastasis-associated lung adenocarcinoma transcript 1 (MALAT1), a long noncoding RNA (lncRNA) of approximately 8,000 nt in length, could counteract the above effects of TGP. MALAT1 can exert competitive endogenous RNA (ceRNA) activity to reduce the gout inflammatory response through the miR-876-5p/NLRP3 pathway (Meng et al., 2021).

### MicroRNAs and other regulatory pathways in gouty arthritis

Acute gout attacks depend on not only activation of the NLRP3 inflammasome but also upregulation of IL-1β transcription. IL-1β is a key proinflammatory cytokine in the inflammatory response of gout, and the mechanism of its proinflammatory effect may be to induce the release of other inflammatory cytokines (e.g., IL-6 and IL-8) through a complex signaling cascade effect, thereby synergistically promoting the occurrence of gout (So and Martinon, 2017; Narang and Dalbeth, 2020; Dalbeth et al., 2021). Studies have shown that miRNAs are involved in the regulation of IL-1β, which in turn plays an important role in GA. MiR-488 and miR-920 can directly bind to the 3′ UTR of IL-1β and target IL-1β (Zhou et al., 2017). Li et al. (2021a) confirmed that miR-221-5p interacts with the 3′ UTR of IL-1β and can target and inhibit IL-1β. An and Yin (2021) verified that miR-192-5p regulates the pathogenesis of GA by targeting EREG protein and inhibiting the activation of M1 macrophages. Jin et al. (2014) found that the miR-155/SHIP-1 pathway leads to upregulation of IL-1β and TNF-α. However, Yang et al. (2018) recently found that miR-155 may be unimportant in MSU-induced gout inflammation in mice, and deletion of miR-155 may not alleviate acute gout inflammation.

## Application of microRNAs in the clinical diagnosis of gouty arthritis

MiRNAs can stably exist in human body fluids, such as serum, urine (Ritter et al., 2020) and saliva (Park et al., 2009). This high stability and the ease of obtaining biological samples suggest that miRNAs may be used for the diagnosis and monitoring of disease processes. Studies have confirmed that in different diseases or different stages of the same disease, the functions and expression profiles of miRNAs *in vivo* have specific characteristics, manifesting as increased or decreased miRNA expression, which is then used as a marker for diagnosing diseases (Alevizos and Illei, 2010). Chen et al. (2019b) found that 16 plasma exosomal miRNAs were specifically expressed in GA patients. The expression of miR-449a was decreased in an MSU crystal-induced GA mouse model (Wang, 2021). MiR-142-3p was highly expressed in both *in vitro* and *in vivo* GA models (Lu et al., 2022). Jin et al. (2014) found that the expression of miR-155 was increased in the SFMC of patients with AGA and a mouse model of gout. In recent years, Zhang et al. (2018) found that the expression of miR-146a was increased in MSU-stimulated mouse bone marrow-derived macrophages. Various other miRNAs, such as miR-488 and miR-920, were also found to be abnormally expressed in GA. Therefore, the abnormal expression of the above miRNAs may provide help for the early diagnosis and treatment of GA and is expected to become a potential biomarker for GA diagnosis.

## Application of microRNAs in the treatment of gouty arthritis with traditional Chinese medicine

At present, the first-line drugs for the clinical treatment of GA include colchicine, nonsteroidal anti-inflammatory drugs, and glucocorticoids (Dalbeth et al., 2019; FitzGerald et al., 2020). However, the clinical use of these drugs has been limited because although they have shown efficacy in the short term, their efficacy still needs to be improved, and the patients are prone to fever, renal, hepatic, and gastrointestinal adverse effects and have narrow treatment windows. In recent years, an increasing number of traditional Chinese medicines have achieved satisfactory results in the treatment of gout because of certain advantages, such as good tolerability, high safety, and few toxic side effects (He et al., 2022). In recent years, it has been found that many Chinese herbal medicines, such as Chuanhutongfeng mixture, Tripterine, Bai Shao, and Noni, are involved in regulating miRNAs, which in turn regulate the related inflammatory response, providing new ideas for the development of new anti-gout drugs (Table 2).

**TABLE 2:** The mechanism of microRNA in the treatment of GA with traditional Chinese medicine.

Chinese Medicines	Effective component	Mechanism	PMID
Chuanhutongfeng mixture	Resveratrol	Chuanhutongfeng mixture upregulated the expressions of miR-486-5p, miR-339-5p, and miR-361-5p and inhibit the expression of proteins CCL2 and CXCL8 in plasma	30854012
Tripterine	Tripterine	Tripterine modulates the miR-449a/NLRP3 axis and the STAT3/NF-κB pathway to alleviate GA.	33559709
Total glucosides of paeony (TGP)	Paeoniflorin	TGP inhibits msu-induced THP-1 macrophage inflammatory response by regulating MALAT1/miR-876-5p/NLRP3 axis and TLR4/MyD88/NF-κB pathway	33677310
Noni	Noni juice	Noni juice could has a certain therapeutic effect on AGA by inhibiting TLR/MyD88/NF-kB pathway	34359507

Chuanhutongfeng mixture is a classic gout treatment, and many research results have confirmed the efficacy and safety of Chuanhutongfeng mixture in the treatment of AGA. One study found that Chuanhutongfeng mixture with sodium alginate was more effective than colchicine in the treatment of AGA (Wang et al., 2015). Wang’s research group found that the clinical efficacy of Chuanhutongfeng mixture in the treatment of AGA was not inferior to that of colchicine, and it had better safety (Wang et al., 2014). Subsequently, their group further improved the formulation of Chuanhutongfeng mixture, and its effectiveness was demonstrated in a mouse model (You et al., 2019). Wang et al. (2019) found that the expression levels of miR-339-5p, miR-486-5p and miR-361-5p were decreased in patients with CGA and that Chuanhutongfeng mixture could upregulate the expression of the above three miRNAs and inhibit the expression of protein chemokine 2 (CCL2) and interleukin 8 (CXCL8), thus exerting a therapeutic effect on CGA.

Noni, also known as *Morinda citrifolia* L., has been used as a medicinal plant for more than 2000 years because of its good medicinal properties and its good preventive and therapeutic effects on acute and chronic diseases such as diabetes, hypertension, and gout (Wang et al., 2002; Palu et al., 2009; Nerurkar et al., 2015). Tahitian Noni Juice (TNJ) was found to downregulate IL-4 expression for immunomodulatory effects (Palu et al., 2008). Palu et al. (2009) subsequently found that the molecular mechanism of TNJ in gout was attributed to its inhibitory effect on xanthine oxidase. Relevant human clinical trials have demonstrated that drinking noni juice is healthy, beneficial and safe and may be closely related to its antioxidant effects (West et al., 2018). Moreover, Li’s group found that miRNAs are also involved in the therapeutic effect of noni juice on AGA. They identified multiple miRNAs expressed abnormally in the colchicine and noni juice groups by modern sequencing techniques and hypothesized that these miRNAs are involved in the regulation of the pathogenesis of AGA in mice (Li et al., 2021b).

## Future prospects

Gout seriously affects normal human life, and miRNAs, as posttranscriptional gene regulators, play important regulatory roles with therapeutic and application potential. A comprehensive review of miRNAs in GA will encourage clinical research and application of miRNAs in the diagnosis and prediction of human disease. At the same time, we should also recognize that the limited data available on the regulatory roles of miRNAs in GA are mainly from mouse models, which may not mimic the complex human situation in all cases, and the lack of clinical data from large samples is not conducive to deepening our understanding of the roles of miRNAs. Therefore, relevant validation not only in animals but also in human clinical trials is needed to further elucidate the relationships between miRNAs and GA.

The ceRNA hypothesis is a gene regulation mechanism in which certain lncRNAs competitively bind to miRNAs, indirectly affecting the regulation of target mRNAs by miRNAs and ultimately affecting gene expression (Salmena et al., 2011). There are approximately 20,000 lncRNAs with potential functions in the human body (Hon et al., 2017). The regulation of lncRNA‒miRNA–mRNA has been demonstrated in various diseases, such as tumors, cardiovascular diseases and autoimmune diseases (Chan and Tay, 2018; He et al., 2018; Mo et al., 2018). With the gradual deepening of ceRNA mechanism research, Hu et al. (2019) found that lncRNA antisense non-coding RNA in the INK4 locus (ANRIL) can upregulate the expression of BRCA1-BRCA2-containing complex subunit 3 (BRCC3) protein by regulating the expression of miR-122-5p in the development of uric acid-induced inflammation. Therefore, the mechanism of action of ceRNA in GA will be worthy of further exploration as the focus of research.

Finally, in addition to Western medicine, existing studies have confirmed that traditional Chinese medicine can treat gout by interfering with the expression of miRNAs. Therefore, the treatment of diseases through miRNAs in traditional Chinese medicine may become a new research direction, and the deepening of research on miRNA mechanisms can provide theoretical support for various clinical therapies, such as traditional Chinese medicine. To improve the therapeutic effect for gout patients, it is also worthwhile to pay extensive attention to the field to determine whether combining treatments can prove the roles of miRNA in gout and provide new diagnostic pathways and therapeutic targets for the clinic, which will open up new potential therapeutic avenues for gout patients.

## Conclusion

This paper systematically summarizes the expression profiles and roles of multiple miRNAs, such as miR-192-5p, miR-221-5p, and miR-920, in the occurrence and development of gout. Targeting miRNAs may be an effective method for the treatment of GA and also provides a possibility for screening new anti-gout drugs. However, the above mentioned miRNAs are only a very small number of known miRNAs. It is believed that with the development of high-throughput whole-genome sequencing technology, more miRNAs that may be involved in the regulation of gout will be discovered, which means that more new therapeutic approaches may be developed.
